# Fast and Accurate Determination of Minute Ochratoxin A Levels in Cereal Flours and Wine with the Label-Free White Light Reflectance Spectroscopy Biosensing Platform †

**DOI:** 10.3390/bios12100877

**Published:** 2022-10-15

**Authors:** Chrysoula-Evangelia Karachaliou, Georgios Koukouvinos, Grigoris Zisis, Dimosthenis Kizis, Evangelia Krystalli, George Siragakis, Dimitris Goustouridis, Sotirios Kakabakos, Panagiota Petrou, Evangelia Livaniou, Ioannis Raptis

**Affiliations:** 1 Immunopeptide Chemistry Lab., Institute of Nuclear & Radiological Sciences & Technology, Energy & Safety, National Centre for Scientific Research “Demokritos”, P.O. Box 60037, 15310 Agia Paraskevi, Greece; 2 Immunoassay/Immunosensors Lab., Institute of Nuclear & Radiological Sciences & Technology, Energy & Safety, National Centre for Scientific Research “Demokritos”, P.O. Box 60037, 15310 Agia Paraskevi, Greece; 3 Institute of Nanoscience and Nanotechnology, National Centre for Scientific Research “Demokritos”, P.O. Box 60037, 15310 Agia Paraskevi, Greece or; 4 Scientific Directorate of Phytopathology, Benaki Phytopathological Institute, 14561 Kifissia, Greece; 5 Yiotis Anonimos Emporiki & Viomixaniki Etaireia, 12131 Athens, Greece; 6 Tuv Austria Food Allergens Labs Ltd., Kalopsidas 38, 7060 Livadia, Cyprus; 7 ThetaMetrisis S.A., Christou Lada 40, 12132 Athens, Greece

**Keywords:** Ochratoxin A, white light reflectance spectroscopy biosensor, immunoanalysis, cereal flours, wine

## Abstract

Ochratoxin A (OTA) is one of the most toxic naturally encountered contaminants and is found in a variety of foods and beverages, including cereals and wine. Driven by the strict regulations regarding the maximum allowable OTA concentration in foodstuff and the necessity for on-site determination, the development of fast and sensitive methods for the OTA determination in cereal flours and wine samples, based on white light reflectance spectroscopy, is presented. The method relied on appropriately engineered silicon chips, on top of which an OTA-protein conjugate was immobilized. A polyclonal antibody against OTA was then employed to detect the analyte in the framework of a competitive immunoassay; followed by the subsequent addition of a biotinylated secondary antibody and streptavidin for signal enhancement. A small size instrument performed all assay steps automatically and the bioreactions were monitored in real time as the software converted the spectral shifts into effective biomolecular adlayer thickness increase. The assay developed had a detection limit of 0.03 ng/mL and a working range up to 200 ng/mL. The assay lasted 25 min (less than 1h, including calibrators/antibody pre-incubation) and was accomplished following a simple sample preparation protocol. The method was applied to corn and wheat flour samples and white and red wines with recovery values ranging from 87.2 to 111%. The simplicity of the overall assay protocol and convenient instrumentation demonstrates the potential of the immunosensor developed for OTA detection at the point of need.

## 1. Introduction

Ochratoxin A (OTA) is a low-molecular-weight compound (403.81 Da) formed as a secondary metabolite by filamentous fungi of the genera *Aspergillus* and *Penicillium* [1]. Due to the colonization of these species into a plethora of crops during cultivation, harvest, and post-harvest storage, OTA has been detected into a series of widely consumed agricultural products [2]. As such, all major cereal grains worldwide, including corn, wheat, maize, oat and barley, have been found to be contaminated with OTA, formed mainly during the storage of grains [3]. Apart from cereals, wine is another edible matrix in which OTA has been detected, originating from fungal contamination of grapes, and subsequently of the grape-must used in the winemaking process [4,5,6,7]. Thus, cereals, along with wine and grape juice, are reportedly the major dietary sources of OTA for humans [3,6].

OTA is considered harmful for humans and animals, since there is evidence associating the consumption of contaminated food with chronic toxicity (genotoxicity, immunotoxicity, nephrotoxicity, hepatotoxicity, etc.), teratogenicity, mutagenicity and carcinogenicity. In accordance with this, the International Agency for Research on Cancer has classified OTA as a group 2B contaminant, i.e., a possible carcinogenic to humans [8,9]. The recognition of the threats that OTA poses in public health is reflected in the stringent regulatory limits set by national and international agencies on the maximum allowable concentration of this toxin in several agricultural products [10,11,12]. Therefore, in order to minimize the risk for public health, the European Union (EU) has established a maximum limit of 3 μg/kg for OTA in cereals and cereal-based products, and 2 ng/mL in grape juice and wine [11], which was adopted by several non-EU countries as well [6].

In compliance with the aforementioned regulatory limits, and in an effort to keep the life-threatening risks posed by OTA to a minimum, various methods for detection and quantification of the toxin have been developed. Nowadays, analysis of OTA in foodstuff is performed mainly by high-performance liquid chromatography (HPLC) coupled to fluorescence or mass spectrometry detectors [13,14]. These analytical methods are characterized by high reliability, selectivity and sensitivity. However, the high analysis cost, the need for skilled personnel and bulky instruments, and the necessity of complex sample treatment consist major bottlenecks, regarding their application to routine high-throughput screening and/or point-of-need analysis of OTA [15]. Immunoanalytical techniques, mainly in the form of enzyme-linked immunosorbent assays (ELISA), constitute another category of methods implemented in OTA determination in food samples due to their simplicity, sensitivity and ability of simultaneous analysis of multiple samples [16]. However, they do not meet the specifications of point-of-need analysis. Currently, the efforts are focusing on two main directions, the development of assays for detection by the naked eye and the development of small-sized instruments based on biosensor platforms [17,18]. Several types of biosensors for OTA detection have been reported in the literature, employing different biorecognition elements, mainly antibodies, aptamers or chelates, as well as different read-out modes, mainly optical and electrochemical [17,19]; however, only a few of them have been tested in the analysis of a broad range of food/beverage samples and/or show the potential of point-of-need application.

**Figure 1:**
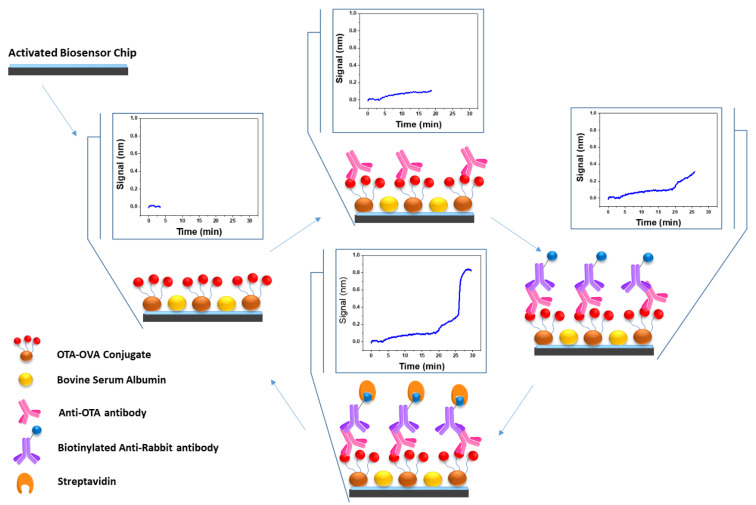
Scheme of assay for OTA determination with the WLRS sensor in the absence of competitor (zero calibrator). The thickness increase in the biomolecular adlayer on top of the sensor chip during the assay caused a shift of the reflectance spectrum towards higher wavelengths. By fitting with the interference equation, the thickness of the adlayer was calculated and interpreted as a signal in the real-time response graphs.

In the current work, a newly developed polyclonal antibody against OTA was employed for the development of an immunosensor for the accurate, rapid, low-cost and laborious-free determination of OTA in cereal flours and wines based on the white light reflectance spectroscopy (WLRS) principle. The WLRS sensing platform has been successfully applied to the quantitative determination of both high- and low-molecular-weight analytes [20] after proper biofunctionalization of the sensing surface with immunoreagents (antigen conjugates or antibodies depending on the assay format). WLRS biosensing principle relies on the real-time monitoring of the reflectance spectrum spectral shifts in the VIS/NIR range and associates them with the effective thickness of the biomolecular layer that grows on the biochip surface during the bioreaction. In particular, the presence of silicon dioxide layer results in an interference spectrum, as the white light that strikes the chip vertically is reflected back by the silicon surface. The increase in biomolecular adlayer thickness onto the chip surface due to binding reactions causes a shift of the interference spectrum towards higher wavelengths. By mathematical processing of the spectra recorded, the effective biomolecular adlayer thickness is determined and presented in real time by the software accompanying the WLRS apparatus. For the specific application of OTA determination in cereal flours and wines, an indirect competitive immunoassay format was implemented. As depicted in Figure 1, an OTA-protein conjugate was immobilized onto the amino-silanized sensor surface and the assay was performed by running mixtures of a rabbit polyclonal antibody against OTA with OTA calibrators or samples. The immunocomplexes formed onto the chip surface were then detected by a biotinylated secondary antibody (goat anti-rabbit IgG antibody) and streptavidin, resulting in significant signal amplification. All assay steps were performed automatically, employing a compact reader that accommodates all optical, fluidic and electronic components needed to perform the assay (Figure S1) and also collects, processes the data, and presents them to the user [21], which demonstrates the potential of the biosensor to eventually serve as a point-of-need method of OTA determination. This approach enabled the determination of OTA within 25 min at concentrations far lower than the maximum acceptable concentration of 3 μg/kg in cereals and 2 ng/mL in wine set by EU. In addition, a quite simple sample preparation procedure was evaluated and successfully applied to alleviate matrix effects. The accuracy of OTA determinations with the proposed methodology was evaluated using cereal flours and white and red wine samples spiked with OTA. Finally, the potential of regeneration and re-use of sensor chips was investigated as a means to reduce the per sample analysis cost.

## 2. Materials and Methods

### 2.1. Reagents and Instrumentation

Ochratoxin A (OTA), fumonisin B1 (FB1), deoxynivalenol (DON), aflatoxin B1 (AFB1), and OTA conjugate with ovalbumin (OTA-OVA) were purchased from Aokin AG (Berlin, Germany). Ochratoxin B (OTB) and ochratoxin C (OTC) were from Cayman Chemical (Ann Arbor, MI, USA). Biotinylated goat anti-rabbit IgG antibody (secondary antibody), streptavidin, polyvinylpyrrolidone (PVP), and (3-aminopropyl)triethoxysilane (APTES) were obtained from Sigma-Aldrich (Darmstadt, Germany). Bovine serum albumin (BSA) was from Acros Organics (Geel, Belgium). IgG Elution buffer was from Thermo Fisher Scientific Inc. (Waltham, MA, USA). All other chemicals were from Merck KGaA (Darmstadt, Germany). RIDASCREEN^®^ Ochratoxin A 30/15 enzyme immunoassay kit was purchased by R-Biopharm AG (Darmstadt, Germany). The water used throughout the study was double distilled. Four-inch Si wafers were purchased from Si-Mat Germany (Kaufering, Germany).

### 2.2. Development and Purification of the Anti-OTA Antibody

The polyclonal anti-OTA antibody was developed in-house. Since OTA cannot elicit antibody development when administered per se in a host-organism (being a “hapten”, i.e., a molecule of low molecular weight), a suitable synthetic OTA-derivative was conjugated to a large carrier protein, i.e., bovine thyroglobulin, and the conjugate was suitably formulated as a water-in-oil (w/o) emulsion in Freund’s adjuvant and, subsequently, used for immunization of two female New Zealand white rabbits. Conjugation to the carrier protein, preparation of the immunogenic emulsions and animal immunization were performed as previously described [22], with slight modifications, mainly concerning the exact time schedule of animal injections/bleedings. The immunization procedure was accomplished at the Animal House of the Institute of Biosciences & Applications, NCSR “Demokritos” (certified installation, EL 25 BIOexp 039, Prefecture of Attica). Animal experimentation was performed in accordance with the Presidential Decree 56/2013 for the Protection of Animals used for Scientific Purposes (Directive 2010/63/EU) and approved by the local committee of the Animal House and the Greek authorities (Prefecture of Attica, Division of Agriculture and Veterinary Medicine (license No. 857573/23-12-2019). Antiserum was received by blood centrifugation at 2000× *g* for 30 min and stored at −30 °C until further processing. The whole IgG fraction was isolated from the antiserum by employing a two-step sequential caprylic acid depletion of serum proteins followed by ammonium sulfate precipitation of immunoglobulins by slightly modifying a published method [23]. In brief, 1 mL of antiserum was mixed with 3 mL of 60 mM acetate buffer, pH 4.0, under gentle vortex. The pH of the solution was first adjusted to 4.5 with addition of 1N NaOH, and then 100 μL of caprylic acid was added drop-wise under continuous stirring for 30 min at room temperature (RT). Then, the mixture was centrifuged for 45 min at 10,000× *g*, the supernatant was collected, filtered through a 0.45 μm filter and its pH was re-adjusted to 7.4 with 1N NaOH. Subsequently, the solution was cooled on ice under vigorous stirring, and 1.1 g of ammonium sulfate was added very slowly. After 30 min, the solution was centrifuged at 10,000× *g* for 30 min at 4 °C, the supernatant was discarded and the pelleted IgGs were re-suspended in 0.01 M phosphate-buffered saline (PBS), pH 7.4. The isolated IgG fraction was finally dialyzed for 72 h against 0.01 M PBS pH 7.4, and its purity was verified by SDS-PAGE. The protein concentration of the IgG fraction was determined using the bicinchoninic acid (BCA) method [24].

### 2.3. Preparation of OTA Calibrators

A 2 mg/mL OTA stock solution was prepared in absolute ethanol, aliquoted and stored at −30 °C. From this solution, calibrators with concentrations ranging from 0.05–200 ng/mL were prepared in 1:9 mixture of absolute ethanol with 10 mM phosphate buffer, pH 7.4, 0.9 % (*w*/*v*) NaCl, 0.02 % (*w*/*v*) KCl, 0.2% (*w*/*v*) BSA (assay buffer). Calibrators were kept aliquoted at −30 °C for up to 2 months.

### 2.4. Foodstuff Treatment

Cereal flours (corn and wheat) were provided by Yiotis S.A. (Athens, Greece) and Food Allergens Laboratories (Livadia, Cyprus). For the extraction of OTA from these samples, the extraction buffer supplied along with the RIDASCREEN^®^ Ochratoxin A 30/15 enzyme immunoassay kit was used according to the manufacturer’s instructions. In brief, 5.0 g of grounded flour sample were added to 25 mL extraction buffer and shaken manually for 5 min. Then, the mixture was centrifuged for 5 min (3500× *g*, RT), and the supernatant was collected and diluted 1:1 (*v*/*v*) with assay buffer, without any sample clean-up or pre-concentration step prior to analysis.

Red and white wines of Greek origin (6 white and 6 red) were purchased from the local market. Bottles were stored at RT and opened just prior to analysis. The pH of each wine sample was first adjusted to 7.4 ± 0.2 with 1N NaOH. White wines were then diluted ten times with assay buffer, whereas red wines were further treated following a simple published protocol [25,26]. In brief, in red wines PVP was added at a final concentration of 3% (*w*/*v*) and the mixture was incubated for 10 min at RT prior to filtration through a 0.45 μm PTFE filter. Finally, the treated red wine samples were diluted ten times with assay buffer.

### 2.5. OTA WLRS Sensor Assay

The FR-Bio reader accommodates the biochips, the optical set-up, the reagents handling module and the electronic module [21]. The optical set-up of the FR-Bio reader includes a stabilized light source that emits in the visible/near infrared spectral range, a custom-made reflection probe, and a miniaturized high-resolution spectrometer. The reflection probe consists of seven fibers with a diameter of 200 microns each; a bunch of six fibers has been arranged at the periphery of the probe, to illuminate the biochip, whereas the seventh one has been placed in the middle of the probe, to collect the reflected light. At the other end, the latter fiber has been coupled to the spectrometer for further analysis of the reflectance spectrum. The reagents handling module includes a carousel that accommodates up to four vials containing the assay reagents required, a programmable micro-pump for supplying the assay solutions at a constant flow rate, and a z-axis moving sampling probe. The reagents-handling module is controlled by an electronics module, which, in addition, controls the spectrometer and the light source operation. The electronics module also provides the communication with the PC, which runs the software, through a standard USB port.

The Si chips were manufactured by thermal oxidation of silicon wafers at 1100 °C, in the clean room facility of the Institute of Nanoscience and Nanotechnology of NCSR “Demokritos”, to grow a 1000 nm thick silicon dioxide layer. The wafers were then diced to chips with dimensions 5 mm × 15 mm, cleaned under sonication in acetone and isopropanol baths and immersed in Piranha solution (1:1 *v*/*v* H_2_SO_4_/30% H_2_O_2_) for 20 min. Then, the chips were washed extensively with distilled water, dried under nitrogen flow, immersed in a 2% (*v*/*v*) aqueous APTES solution for 20 min and cured for 20 min at 120 °C.

Chips were kept at RT in a desiccator for at least 48 h prior to spotting a 200 μg/mL OTA-OVA conjugate solution in 50 mM carbonate buffer, pH 9.25. After overnight incubation at RT, the spotted chips were rinsed with PBS and then blocked via immersion for 3 h in 2% (*w*/*v*) BSA solution in PBS. After that, the chips were again washed with PBS, dried under N_2_ and assembled to a custom-designed microfluidic cell (Jobst Technologies GmbH, Freiburg, Germany). The so prepared biochips were inserted in the docking station of FR-Bio reader and connected by tubing to the vials containing the assay reagents and the pump, set at a constant rate of 50 μL/min. The docking station of the FR-Bio reader was then placed under the reflection probe and the protocol sequence was initiated by the software.

At first, assay buffer run for 3 min to acquire a stable baseline. Τhen, 1:1 volume mixtures (pre-incubated for 30 min at RT) of a 1 μg/mL rabbit anti-OTA antibody solution in assay buffer with calibrators (0.05–200 ng/mL OTA in assay buffer) or cereal flour extracts (2 times diluted with assay buffer) or wine samples (10 times diluted with assay buffer) were passed over the chip for 15 min. After that, a biotinylated anti-rabbit IgG antibody (1:200 diluted in assay buffer) run for 5 min, followed by a 10 μg/mL streptavidin solution in assay buffer for 3 min. Finally, the chip was regenerated by passing a 0.1 M glycine-HCl solution, pH 2.5 (IgG elution buffer), for 4 min, followed by equilibration with assay buffer. A schematic of the assay procedure is provided in Figure 1. The calibration curve was created by plotting the effective biomolecular layer thickness (signal, S), corresponding to different calibrators (S_x_) expressed as percentage of the zero calibrator’s signal (maximum signal; S_0_) against the OTA concentration in the calibrator solutions.

## 3. Results and Discussion

### 3.1. Development of the OTA Assay

#### 3.1.1. Selection of Immunoassay Format

**Figure 2:**
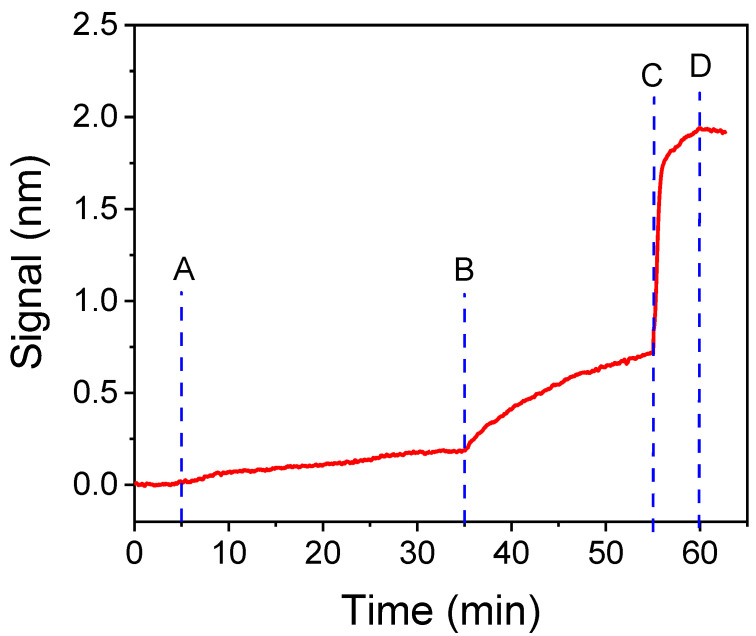
Real-time signal obtained from a chip functionalized with OTA-OVA conjugate (200 μg/mL) upon running: assay buffer (start to point A); a 1:1 (*v*/*v*) mixture of zero calibrator with a 1.0 μg/mL rabbit anti-OTA antibody solution (A,B); a 1:200 dilution of biotinylated secondary antibody (B,C); and a 10 μg/mL streptavidin solution (C,D).

OTA is a low-molecular-weight analyte and, therefore, its immunochemical determination is accomplished following a competitive immunoassay format, based on either the immobilization of the anti-OTA specific antibody or an OTA-protein conjugate. For the development of the OTA assay on the WLRS sensor, the second approach was favored, mainly because antigen-protein conjugates are more stable under storage conditions and for long-term use, as opposed to the respective antibodies. After the primary immunoreaction, i.e., the competitive reaction between the OTA in the calibrators/samples and the OTA immobilized onto the chip surface as a protein conjugate for binding to the anti-OTA antibody, additional steps might be employed in order to amplify the signal. Although these additional steps prolong the assay, they allow the obtaining of higher absolute signals using lesser amounts of antigen-specific antibody, which is one of the main factors affecting the assay sensitivity and cost. In the current work, the primary immunoreaction was followed by two additional steps, first with a biotinylated goat anti-rabbit IgG antibody (secondary antibody), and second, streptavidin. In Figure 2, the signal evolution in real-time over the course of primary and secondary immunoreaction and the subsequent reaction with streptavidin is presented. After 30 min, the primary immunoreaction gave a signal (effective thickness of the biomolecular layer) of approximately 0.18 nm, whereas 20 min reaction with the secondary antibody provided an additional signal of 0.54 nm (3-fold increase, compared to primary immunoreaction). An additional 5 min reaction with streptavidin further increased the signal by 1.22 nm (1.7-fold increase, compared with both primary and secondary immunoreaction; 6.7-fold increase, compared to primary immunoreaction). Overall, the signal increased more than 10 times, compared with that received by the primary immunoreaction when the two signal amplification steps were implemented. The significant signal amplification achieved by the additional reaction steps is ascribed to the binding of more than one secondary antibody molecule per molecule of primary antibody and of more than one streptavidin molecules per molecule of biotinylated secondary antibody. Since the WLRS detection principle relies on the determination of biomolecular layer thickness, the additional reaction steps, which apparently result in the accumulation of multiple layers of protein molecules on the sensing surface, allow for the easier detection of signal differences, arising from the primary immunoreaction. Thus, the 3-step assay was selected and further experimentation focused on reducing the overall assay time. As shown in Figure 2, the signal during the primary immunoreaction increased linearly with the reaction time, whereas the reaction with the secondary antibody tends to saturate after 20 min, and the reaction with streptavidin is almost completed (95% of the plateau signal) in 3 min. Keeping the second and the third step duration at 10 and 3 min, respectively, the zero calibrator signal values for primary immunoreaction duration of 5, 10, 15 and 20 min were determined. As shown in Figure S2, the overall signal was reduced by approximately 20% for primary immunoreaction duration of 15 min instead of 30 min. On the other hand, for primary immunoreaction duration of 15 min and 3 min reaction with streptavidin, the signal was reduced by approximately 40% when the secondary immunoreaction duration was reduced from 20 to 5 min (Figure S3). Thus, reduction in the whole assay duration from 55 to 25 min resulted in a signal decrease of about 50%. Nonetheless, the signal received under these conditions (approximately 1 nm) was adequate taking into account that the baseline signal variation was less than 5%.

#### 3.1.2. Optimization of Assay Parameters

**Figure 3:**
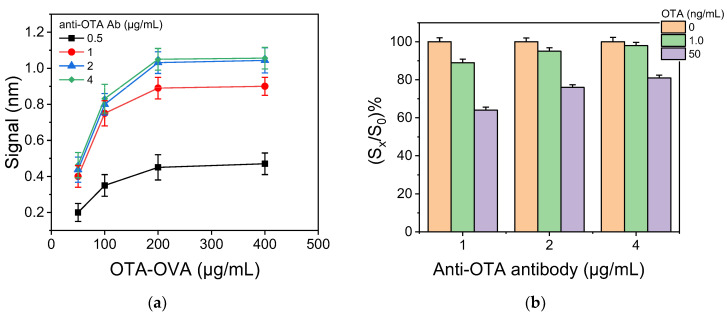
(**a**) Effect of OTA-OVA concentration used for chip coating on the zero calibrator signal received for anti-OTA antibody concentrations of 0.5 (black squares), 1.0 (red circles), 2.0 (blue triangles), or 4.0 μg/mL (green diamonds). The primary immunoreaction duration was 15 min. Each point is the mean value of three measurements ± SD. (**b**) Effect of anti-OTA antibody concentration on the percent signal values obtained for calibrators, containing 1.0 (green columns) and 50 ng/mL OTA (purple columns), with respect to zero calibrator (orange columns). The chip was coated with a 200 μg/mL OTA-OVA solution. Each point is the mean value of three measurements ± SD.

The absolute zero calibrator signal and the sensitivity of a competitive immunoassay, as determined by the slope of the calibration curve, depend mainly on the protein conjugate concentration (solid-phase antigen) used for coating of the chips and the concentration of analyte-specific antibody. To determine the optimum combination of these two parameters for the OTA assay, concentrations of the OTA-OVA conjugate, ranging from 50 to 500 μg/mL were used for coating the chips, which were then assayed with anti-OTA antibody concentrations, ranging from 0.5 to 4 μg/mL. As shown in Figure 3a, maximum plateau zero calibrator signal values were obtained with OTA-OVA conjugate concentrations equal to or higher than 200 μg/mL for all antibody concentrations tested; thus, this concentration was selected for further experiments. On the other hand, plateau zero calibrator signals were obtained for concentrations of the anti-OTA antibody equal to or higher than 2 μg/mL (Figure 3a). However, as can be deduced by the data presented in Figure 3b, the assay sensitivity was significantly improved when a 1 μg/mL antibody solution was used. In an attempt to further improve the assay sensitivity, chips coated with a 100 μg/mL OTA-OVA conjugate were tested using the anti-OTA antibody at a concentration of 1 μg/mL. This combination provided a zero calibrator signal of 0.75 nm without significantly improving the percent signal drop achieved in presence of OTA (Figure S4).

Therefore, 200 μg/mL OTA-OVA concentration in combination with 1 μg/mL anti-OTA antibody was adopted in the final protocol, as the best compromise between assay sensitivity and absolute value of zero calibrator signal.

The incubation of calibrators/samples with the anti-OTA antibody prior to introduction onto the biochip was investigated as a means to improve the assay sensitivity by favoring the reaction of the antibody with OTA in calibrators/samples over OTA immobilized onto the chip surface in the form of OTA-OVA conjugate. As depicted in Figure S5, pre-incubation dramatically enhanced the sensitivity of the assay, even for the shortest time tested (10 min). Optimal results were obtained for pre-incubation equal to or longer than 30 min, and, therefore, a 30 min pre-incubation step was incorporated into the assay protocol.

**Figure 4:**
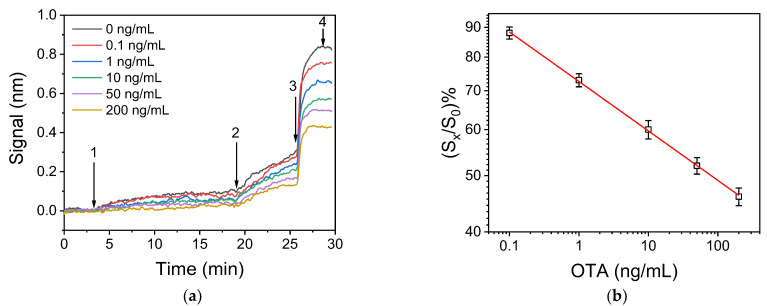
(**a**) Real time responses obtained from biochips functionalized with 200 μg/mL OTA-OVA conjugate upon running: assay buffer (start to arrow 1); a 1:1 *v*/*v* mixture of OTA calibrators (0–200 ng/mL) with a 1 μg/mL anti-OTA antibody solution (arrows 1–2); a 1:200 dilution of biotinylated anti-rabbit IgG antibody (arrows 2–3); and a 10 μg/mL streptavidin solution (arrows 3–4). (**b**) Typical linearized calibration curve obtained with OTA calibrators in assay buffer. Each point represents the mean value of four runs ± SD.

The real-time responses obtained for OTA calibrators with concentrations ranging from 0 to 200 ng/mL using the selected assay conditions are provided in Figure 4a, while in Figure 4b, a typical linearized calibration curve is shown. The linear regression equation was as follows:

log(y) = −0.0852 (±0.0009) × log(x) + 1.860 (±0.001),

where y is the percent signal value, with respect to zero calibrator value and x the OTA concentration. The coefficient of determination value was R^2^ = 0.995.

The analytical sensitivity of the developed immunosensor was evaluated through the calculation of the detection (LoD) and quantification limit (LoQ). The LoD was calculated as the OTA concentration, corresponding to percent signal value, equal to 100-3SD of 12 measurements of zero calibrator, while the LoQ was calculated as the OTA concentration, corresponding to percent signal value, equal to 100-6SD of 12 measurements of zero calibrator. The percent SD of these 12 measurements was 2.2% (Table S1), and thus the LoD and LoQ values were determined to be 0.03 and 0.06 ng/mL, respectively, based on the calibration curve of Figure 4b. The assay dynamic range was extended up to 200 ng/mL. The intra-assay coefficient of variation (CV) was assessed by four replicate measurements of each calibrator in the same run and was less than 5.0%, while the inter-assay CV, as assessed by five measurements of calibrators in five consecutive days, was less than 9.0%.

### 3.2. Evaluation of Rabbit Anti-OTA Antibody Specificity

The specificity of the in-house developed rabbit antibody against other mycotoxins that could be present in food/beverages, e.g., wine [7], was determined though cross-reactivity studies. The mycotoxins tested were the ochratoxin B (OTB) and ochratoxin C (OTC), which along with OTA form the ochratoxin group, as well as deoxynivalenol (DON), fumonisin B1 (FB1) and aflatoxin B1 (AFB1)—each of the latter three toxins is the main representative of three mycotoxin groups (trichothecenes, fumonisins and aflatoxins, respectively) usually detected in cereal and grape products [27]. For this purpose, solutions of each potential cross-reactant mycotoxin with concentrations ranging from 0.1 to 20,000 ng/mL were assayed using a chip coated with OTA-OVA conjugate after 30 min pre-incubation with the anti-OTA antibody. Percent cross-reactivity (%CR) was calculated as follows:

% CR = (IC_50_ target analyte/IC_50_ cross-reactant) × 100

where IC_50_ target analyte and IC_50_ cross-reactant are the concentrations of OTA and putative cross-reactant, respectively, which cause 50% inhibition of the zero calibrator’s signal. The chemical structures of the compounds checked as putative cross-reactants and the respective %CR values determined are presented in Table 1.

As shown, OTB—a dechloro derivative of OTA (Table 1)—exhibits a cross-reactivity of 0.4%, whereas OTC—the ethyl ester of OTA (Table 1)—exhibits a cross reactivity of 43%. The low cross-reactivity value shown by the anti-OTA antibody for OTB may indicate involvement of the chlorine atom of OTA in the formation of the hapten-epitope. On the other hand, the rather high cross-reactivity value shown for OTC may be attributed to the strikingly structural similarity of OTC with OTA (Table 1); this value is not, however, considered discouraging, since OTA has been well documented as the most abundant and most toxic member of the ochratoxin group [28], compared with both OTC and OTB. In support of this, it should be commented here that commercially available ELISA kits for quantitative determination of OTA used by analytical laboratories worldwide, such as the one used in the current study, i.e., RIDASCREEN^®^ Ochratoxin A 30/15 enzyme immunoassay kit, report similar cross-reactivities with OTB and OTC [29].

The cross-reactivity of the anti-OTA antibody for DON, FB1 and AFB1, which differ structurally from OTA but they may co-exist with it in food/beverage samples, was not detectable (ND), even in the highest concentration tested (20,000 ng/mL). These results indicate high specificity of the anti-OTA antibody.

### 3.3. Optimization of the Sample Preparation Procedure

**Figure 5:**
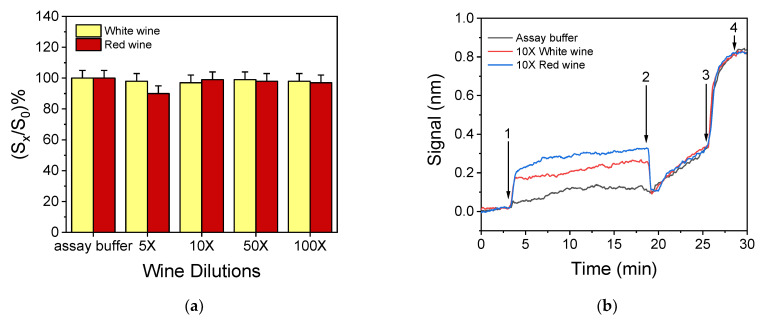
(**a**) Effect of white (yellow columns) and red wine (red columns) dilutions with assay buffer on the zero calibrator signal, with respect to signal in buffer. Each bar represents the mean value of three measurements ± SD. (**b**) Real-time responses obtained from a chip functionalized with OTA-OVA conjugate (200 μg/mL) upon running: arrow 1 to 2, a mixture of zero calibrator prepared in assay buffer (black line) or 10-time diluted white wine (red line) or red wine (blue line) with 1 μg/mL of rabbit anti-OTA Ab (1:1 *v*/*v*); arrow 2 to 3, biotinylated secondary antibody; and arrow 3 to 4, streptavidin.

Regarding the analysis of cereal flour samples, it was found by analyzing eight wheat and six corn flour samples (Table S2), that the presence of extraction buffer did not affect the assay performance once the extract has been diluted 2-fold with assay buffer. On the other hand, wine was found to affect the antibody–antigen binding possibly due to its acidic pH and the phenolic compounds it contains, the latter in higher concentration in red rather than in white wines. Thus, a sample treatment procedure able to alleviate the matrix effect on the performance of the immunoassay had to be established using wines that had been analysed with a commercially available enzyme immunoassay kit (RIDASCREEN^®^, R-Biopharm) and found not to contain detectable concentrations of OTA. The treatment selected was based on already published protocols [25,26] and included wine neutralization by addition of 1 N NaOH solution. The volume of NaOH solution required to achieve this neutralization did not practically change the sample volume. For white wines, this treatment, followed by dilution with assay buffer, was proved adequate to alleviate any matrix effect (Figure 5a, yellow columns) even for the lowest dilution tested (5-fold). For red wines, however, further treatment was necessary. Thus, after neutralization, PVP was added and a 10 min incubation followed by filtration was applied to remove polyphenols. Finally, dilution with assay buffer was performed and the least dilution required to alleviate matrix effects was determined. As shown in Figure 5a for red wines (red columns), a 10-fold dilution with assay buffer was required to suppress any matrix effect on the zero calibrator’s signal. Since this dilution also worked for white wines, it was included in the protocol for wine sample preparation prior to analysis with the WLRS sensor. Figure 5b presents the real-time responses, corresponding to a zero calibrator prepared in assay buffer, as well as to white and red wine processed, as described above, and diluted 10 times with assay buffer. As shown, the introduction of the wine sample/antibody mixture caused an increase in the signal (arrow 1 tο 2) that was more pronounced for the red wine. This effect, however, ceased when the secondary antibody solution was introduced (arrow 2 to 3). These abrupt changes in the sensor response could be attributed to differences in the refractive index between the buffer and the diluted white and red wine, as well as to the presence of colored substances, especially in red wine. As mentioned, upon removal of the wine-containing solutions, the effect ceased to exist and the responses obtained for both the buffer and the diluted wine samples during the secondary immunoreaction and the reaction with streptavidin are identical, indicating the absence of the matrix effect. These results were confirmed using six white and six red wines of Greek origin made from different grape varieties. The zero calibrator signals obtained for these wines are provided in Table S3 of Supplementary Material. The absence of any matrix effect was also confirmed by the fact that almost superimposable calibration curves were obtained with calibrators prepared either in assay buffer or in white and red wines, as described in Section 2.4 (Figure S6).

### 3.4. Accuracy and Precision of the Developed Sensor

The accuracy of measurements performed with the developed immunosensor was evaluated through recovery experiments. To this end, two cereal flour samples—one wheat and one corn—and two wine samples—one white and one red—were analyzed with a commercially available enzyme immunoassay kit (RIDASCREEN^®^, R-Biopharm), prior to the addition of OTA. All samples were found not to contain detectable concentrations of OTA; thus, they were fortified with addition of OTA at three concentration levels, i.e., 5, 25 and 60 ng/mL. The samples were treated as described in Section 3.3. and subsequently analyzed in triplicate with the WLRS sensor. The recovery values, calculated as the ratio of the OTA amount determined (taking into account the dilution factor applying to different samples) to that actually added in the sample are presented in Table 2. As shown, the recovery values obtained ranged from 87.2–111%, demonstrating good accuracy of the OTA WLRS-immunosensor.

The reproducibility of the WLRS OTA assay was assessed by running the spiked samples prepared either in white wine (5, 25 and 60 ng/mL) or in corn flour (5, 25 and 60 ng/mL) in triplicate within the same day and in 10 different days in order to calculate the intra- and inter-assay coefficients of variation, respectively. The intra-assay CV values were ≤5.4, and the inter-assay CV values ≤7.2%.

Moreover, three corn flour samples that were found to contain different OTA concentrations when analysed following an LC–MS/MS method [30] were also analysed with the WLRS sensor. As shown in Table 3, the results obtained with the immunosensor were approximately 13 to 20% higher than those obtained with the LC-MS/MS method. This could be ascribed to the fact that the antibody involved in the study recognizes, to some extent, other ochratoxins that could be present in the samples (Section 3.2).

### 3.5. Regeneration and Reuse of Chips

**Figure 6:**
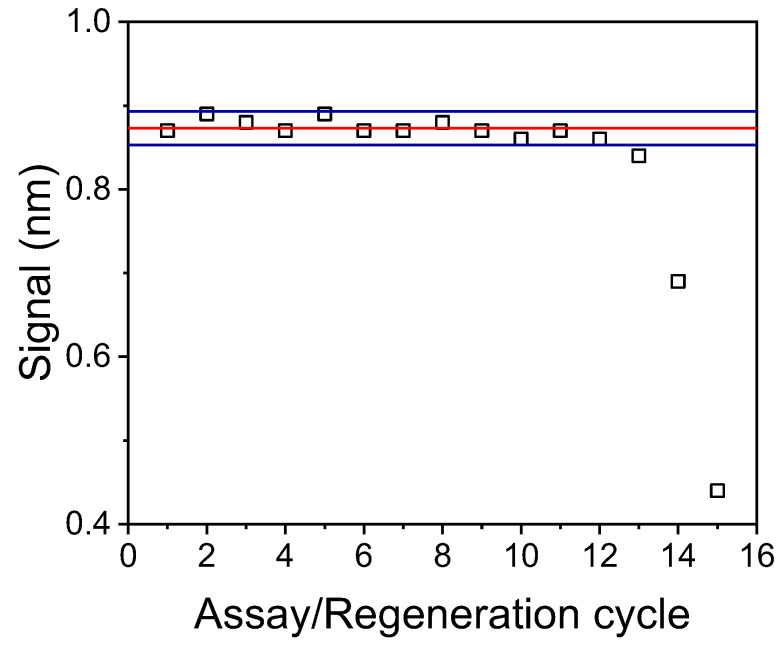
Responses obtained from a single chip for 15 consecutive assay/regeneration cycles. Red line corresponds to the mean value of the first 12 cycles and blue lines to the mean value ± 2SD limits.

The stability of the developed immunosensor response to sequential assay/regeneration cycles was also determined to exploit the possibility to use a single biofunctionalized chip for the analysis of several samples, and thus reduce the per sample analysis cost. The regeneration was achieved by running a 0.1 M glycine-HCl buffer, pH 2.5, for 3 min after completion of the assay. Figure 6 shows the responses obtained for zero OTA calibrator from a single biochip in 15 assay/regeneration cycles performed over three days. As clearly shown, for up to 12 assay/regeneration cycles, all values consistently fall within the mean value ± 2SD range, demonstrating the potential of biosensor reuse.

### 3.6. Comparison with Other OTA-Biosensors

In the last decade, OTA has received particular attention due to its serious effects in human health, and thus a great deal of effort from researchers all over the world has been devoted to the development of biosensors for OTA determination in various food and drink commodities, including cereals and wine [17,18]. The majority of the reported OTA biosensors are optical and use specific labels, e.g., fluorescent ones, whereas only few of them are label-free, including SPR-based sensors for OTA. Most of the SPR-based sensors for OTA employ antibodies as biorecognition element [26,31,32,33,34,35], while a few of them are aptamer-based [36,37,38]. Among the antibody-based OTA sensors, two sensors employ antibodies “loaded” to gold nanoparticles in order to amplify the signal, and thus achieve LoDs fulfilling the EU requirements; one of them achieved a LoD of 0.19 ng/mL in wine, but the assay duration is two times longer than that of the proposed WLRS-immunosensor [26], and the other one achieved an LoD of 0.4 ng/mL in wine, and 0.3 and 0.5 ng/g in oat and corn, respectively, with an analysis time of less than 10 min [35]. An aptamer-based SPR sensor has also been employed for the determination of OTA in red wine [36], claiming a detection limit of 5 pg/mL for an assay duration of 5 min; however, a complicated extraction method with organic solvent is employed that takes at least one day to be completed. Compared to SPR-based OTA-sensors, which do not employ labels [31,32,33,34], the proposed one is still more sensitive (3 to 23 times more sensitive), although less fast (two to six times longer duration). Another SPR-related and label-free sensing principle that has been implemented for OTA determination is the one relying on localized SPR (LSPR), in which the sensing surface consists of gold nanoparticles instead of a continuous gold layer. There are two literature references regarding OTA detection with LSPR sensors both employing aptamers as recognition elements. The first is based on a planar substrate [37] and reports a LoD similar to that of the proposed WLRS-immunosensor, while in the second one the gold nanoparticles are deposited on an optical fiber [38], achieving an impressive LoD of 2 nM (9.6 pg/mL) in grape juice. In both cases, the assay duration was comparable to that of the proposed WLRS-sensor. The detection principle, LoD, dynamic range, assay duration and sample type tested for each of the above discussed label-free sensors are summarized in Table 4.

The immunosensor proposed herein is the first optical sensor using “compact” instrumentation that is based on the white light reflectance spectroscopy and has, therefore, high potential to evolve to a point-of-need analytical tool. The same detection principle with less integrated instrumentation has been previously applied for the determination of other mycotoxins in different matrices, including aflatoxin B1, fumonisins B and deoxynivalenol in cereals and aflatoxin M1 in milk [20], but not to the detection of OTA. In terms of analytical sensitivity, the LoD of the proposed method (30 pg/mL in buffer, 60 pg/mL in cereal flour extracts, which corresponds to 0.3 μg/kg in the initial cereal sample and 300 pg/mL in wine) is 10 times lower than the current EU regulatory limit for OTA in cereals (3 μg/kg) and 7 times lower than the one in wines (2 ng/mL). Overall, in comparison with other recently reported OTA-sensors, the WLRS-immunosensor can be considered a highly competitive and promising analytical device for OTA determination in food/beverage samples.

## 4. Conclusions

A label-free immunosensor based on white light reflectance spectroscopy and run on a small size compact reader for determination of OTA in cereals and wine samples has been presented. The advantages of the proposed immunosensor are the short analysis time (25 min assay plus 30 min pre-incubation of samples/calibrators with the anti-OTA antibody), the simple sample preparation procedure and the excellent analytical performance, both in terms of analytical sensitivity (LoD: 0.030 ng/mL in buffer, 0.060 ng/mL in cereal flour extract and 0.3 ng/mL in wine) and the accuracy/reproducibility of the measurements. Additionally, the proposed immunosensor could be regenerated and reused at least 12 times, without the loss of its analytical performance. Taken altogether, the excellent analytical performance of the newly developed immunosensor and the relatively small size of the reader, hold great promise for future application at the point of need. Thus, the implementation of the developed sensor could facilitate the regular monitoring of OTA levels in food/beverage matrices from production to shelf, guaranteeing the protection of public health from the adverse effects originating from exposure to this extremely hazardous mycotoxin.

## Figures and Tables

**Table 1:** Cross reactivity shown by structurally related- and unrelated-to-OTA toxins.

Toxin	Structure	IC_50_ (ng/mL)	% CR
Ochratoxin A	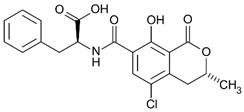	75	100
Ochratoxin B	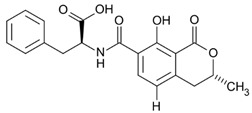	18,750	0.4
Ochratoxin C	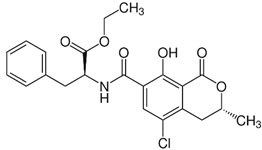	173	43.3
Deoxynivalenol	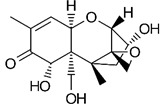	ND	-
Aflatoxin B1	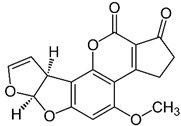	ND	-
Fumonisin B1	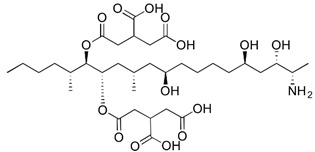	ND	_-_

**Table 2:** Percent recovery values of OTA spiked in cereal flours and white and red wine samples.

Food Sample	Spiked Concentration (ng/mL)	Determined Concentration (ng/mL)	% Recovery
Wheat flour	5	5.4 ± 0.3	108
	25	23.9 ± 1.5	95.6
	60	61.1 ± 3.0	102
Corn flour	5	4.6 ± 0.1	92.0
	25	22.3 ± 1.1	89.2
	60	57.6 ± 2.8	96.0
White wine	5	4.9 ± 0.2	98.0
	25	26.4 ± 1.0	106
	60	54.2 ± 2.2	90.3
Red wine	5	5.6 ± 0.2	111
	25	26.3 ± 1.3	105
	60	52.3 ± 2.5	87.2

**Table 3:** Concentrations of OTA determined in corn flour samples by the developed immunosensor and the reference LC-MS/MS method.

Sample Number	LC-MS/MS (μg/kg)	WLRS Immunosensor (μg/kg)	% Deviation
1	7.44	8.9 ± 0.42	19.6
2	1.02	1.2 ± 0.08	17.6
3	11.6	13.1 ± 0.65	12.9

**Table 4:** Comparison of the WLRS-immunosensor developed for OTA determination with other OTA label-free optical sensors reported in the literature.

Sensing Principle	Recognition Element	Sample Type	LOD	Dynamic Range	Assay Duration	Reference
WLRS	antibody	buffer	0.03 ng/mL	0.06–200 ng/mL	25 min	this work
		Cereals (corn, wheat)	0.3 ng/g	0.6–2000 ng/g		
		wine	0.3 ng/mL	0.6–2000 ng/mL		
SPR	antibody	wine	0.19 ng/mL	0.68–100 ng/mL	55 min	[26]
SPR (6-plex)	antibody	barley	3.0 ng/g	13–320 ng/g	4 min	[31]
SPR (2-plex)	antibody	beer	7.0 ng/mL	10–120 ng/mL	7 min	[32]
SPR	antibody	corn/wheat	1.27 ng/mL	1.98–28.22 ng/mL	13 min	[33]
SPR	antibody	white wine	0.94 ng/mL in buffer	1–50 ng/mL in buffer	-	[34]
SPR	antibody	oat, corn, wine, grape juice, apple juice	0.3 ng/g in oat 0.5 ng/g in corn 0.4 ng/mL in wine	1–100 ng/mL ^a^	<10 min	[35]
SPR	aptamer	red wine, peanut oil	0.005 ng/mL in buffer	0.094–10 ng/mL in buffer	5 min	[36]
LSPR	aptamer	corn	<0.4 ng/g	0.4–40 ng/g	<20 min	[37]
fiber-optic LSPR	aptamer	grape juice	0.0096 ng/mL	0.0096–96 ng/mL	<30 min	[38]

^a^: estimated by the calibration curves presented in Ref. [34].

## Data Availability

The data presented in this study are available on request from the corresponding author. The data are not publicly available due to privacy issues.

## Supplementary Materials

The following supporting information can be downloaded at: https://www.mdpi.com/article/10.3390/bios12100877/s1, Figure S1: Picture of the WLRS instrument set-up used for OTA determination; Figure S2: Effect of primary immunoreaction duration to zero calibrator signals obtained for a 10-min secondary immunoreaction and 3-min reaction with streptavidin. Each point is the mean of three measurements ± SD; Figure S3: Effect of secondary immunoreaction duration to zero calibrator signals obtained for a 30-min primary immunoreaction and 3-min reaction with streptavidin. Each point is the mean of three measurements ± SD; Figure S4: Effect of OTA-OVA conjugate concentration on the signal values obtained for calibrators containing 1.0 (green columns) and 50 ng/mL OTA (purple columns) with respect to zero calibrator (orange columns). The anti-OTA antibody concentration was 1 μg/mL. Each point is the mean value of three measurements ± SD; Figure S5: Effect of pre-incubation of anti-OTA antibody with OTA calibrators on assay sensitivity. Calibration curves obtained when the mixtures of the anti-OTA antibody with OTA calibrators were pre-incubated for 10 (red circles), 30 (blue up triangles), 60 (green down triangles) or 120 min (orange diamond). The calibration curve obtained without pre-incubation is also included (black squares). Each point is the mean value of three measurements ± SD; Figure S6: Calibration curves obtained with OTA calibrators prepared in assay buffer (black squares), white wine treated and 10-times diluted (red circles) or red wine treated and 10-times diluted (blue triangles). Each point is the mean value of three measurements ± SD. Table S1: Zero calibrator signal values obtained from 12 measurements for the determination of SD and, consequently, assay LoD and LoQ; Table S2: Zero calibrator signal values obtained from 8 wheat flour, 3 corn starch and 3 corn flour samples after extraction and 2-fold dilution; Table S3: Zero calibrator signal values obtained from 6 white wine and 6 red wine after 10-times dilution.
