# Supplementary material for: Fast and Accurate Determination of Minute Ochratoxin A Levels in Cereal Flours and Wine with the Label-Free White Light Reflectance Spectroscopy Biosensing Platform
Source: Biosensors (Basel). 2022 Oct 15;12(10):877. doi: 10.3390/bios12100877 (PMC9599867; doi:10.3390/bios12100877)
Supplement: Supplementary file 1 [file biosensors-12-00877-s001.zip › biosensors-1922266-supplementary.pdf]

biosensors

MDPI

Supplementary Information

Article

Fast and Accurate Determination of Minute Ochratoxin A Levels in Cereal Flours and Wine with the Label-Free White Light Reflectance Spectroscopy Biosensing Platform

Chrysoula-Evangelia Karachaliou [1,*], Georgios Koukouvinos [2], Grigoris Zisis [3], Dimosthenis Kizis [4], Evangelia Krystalli [5], George Siragakis [6], Dimitris Goustouridis [7], Sotirios Kakabakos [2], Panagiota Petrou [2,*], Evangelia Livaniou [1] and Ioannis Raptis [3,7]

[1] Immunopeptide Chemistry Lab, Institute of Nuclear & Radiological Sciences & Technology, Energy & Safety, National Centre for Scientific Research ''Demokritos'', P.O. BOX 60037, 15310 Agia Paraskevi, Greece
[2] Immunoassay/Immunosensors Lab, Institute of Nuclear & Radiological Sciences & Technology, Energy & Safety, National Centre for Scientific Research ''Demokritos'', P.O. BOX 60037, 15310 Agia Paraskevi, Greece
[3] Institute of Nanoscience and Nanotechnology, National Centre for Scientific Research ''Demokritos'', P.O. BOX 60037, 15310 Agia Paraskevi, Greece
[4] Scientific Directorate of Phytopathology, Benaki Phytopathological Institute, 14561 Kifissia, Greece
[5] Yiotis Anonimos Emporiki & Viomixaniki Etaireia, 12131 Athens, Greece
[6] Tuv Austria Food Allergens Labs Ltd, Kalopsidas 38, 7060 Livadia, Cyprus
[7] ThetaMetrisis S.A., Christou Lada 40, 12132 Athens, Greece
* Correspondence: xrisak15@hotmail.com (C.-E.K.); ypetrou@rrp.demokritos.gr (P.P.)

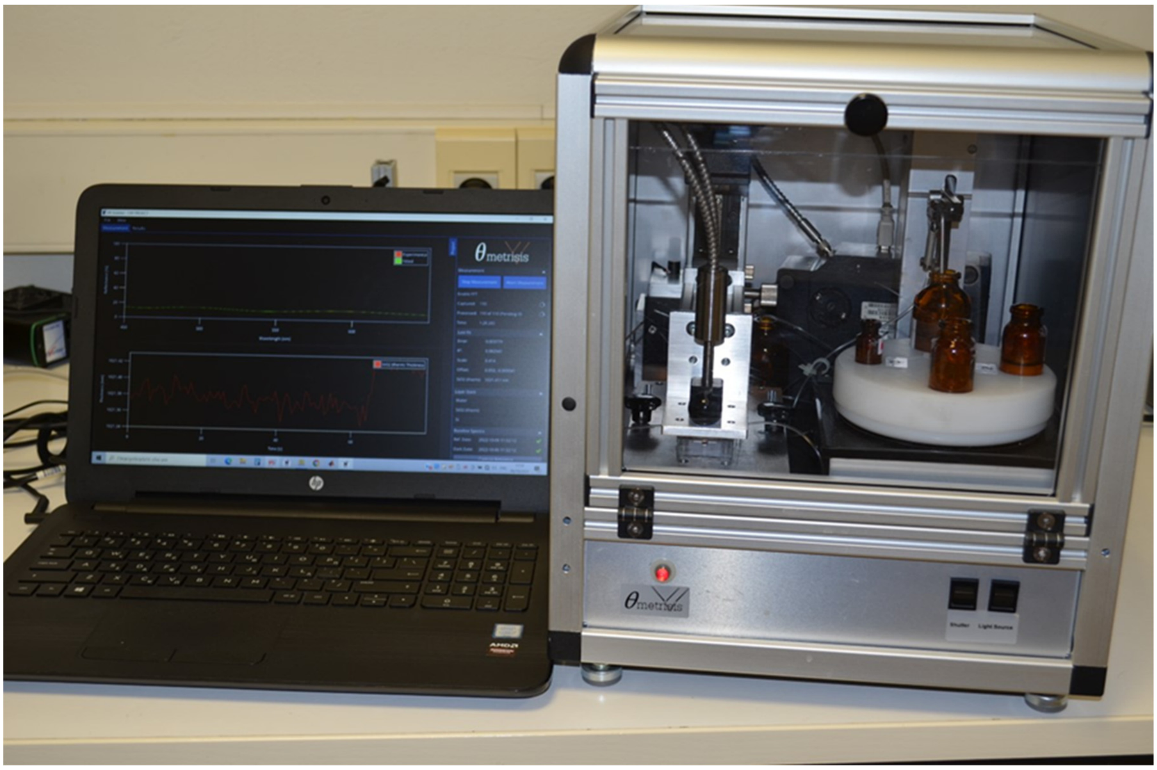

Figure S1. Picture of the WLRS instrument set-up used for OTA determination.

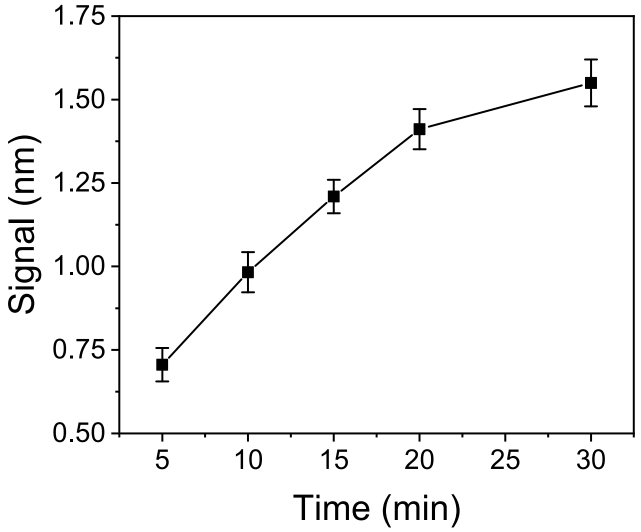

Figure S2. Effect of primary immunoreaction duration to zero calibrator signals obtained for a 10-min secondary immunoreaction and 3-min reaction with streptavidin. Each point is the mean of three measurements ± SD.

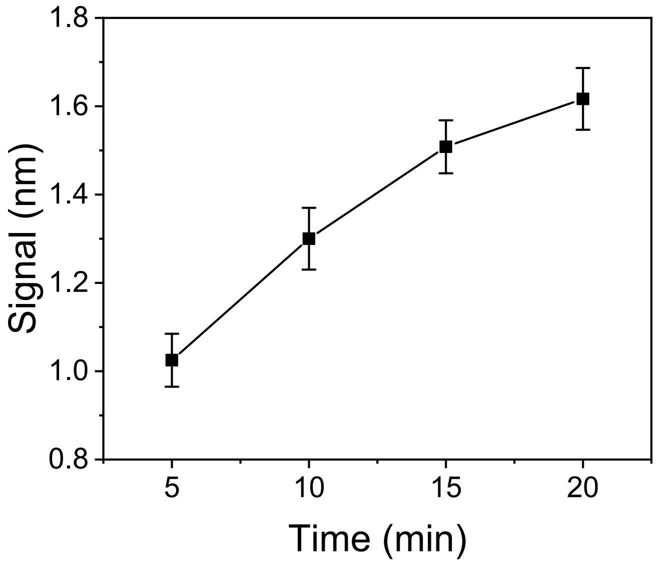

Figure S3. Effect of secondary immunoreaction duration to zero calibrator signals obtained for a 30-min primary immunoreaction and 3-min reaction with streptavidin. Each point is the mean of three measurements ± SD.

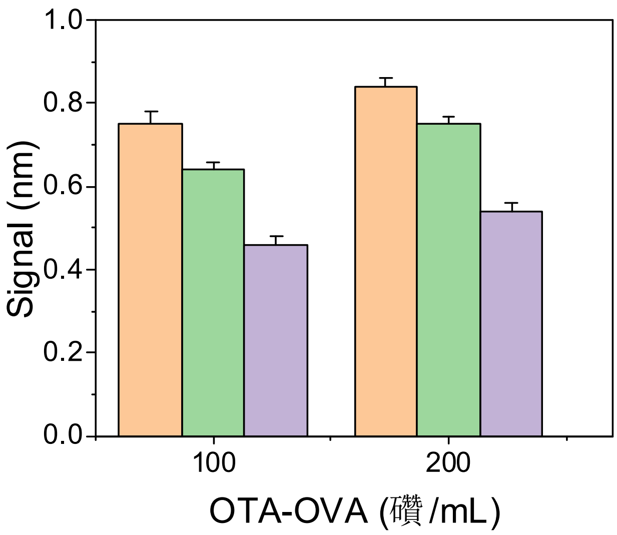

Figure S4. Effect of OTA-OVA conjugate concentration on the signal values obtained for calibrators containing 1.0 (green columns) and 50 ng/mL OTA (purple columns) with respect to zero calibrator (orange columns). The anti-OTA antibody concentration was 1 μg/mL. Each point is the mean value of three measurements ± SD.

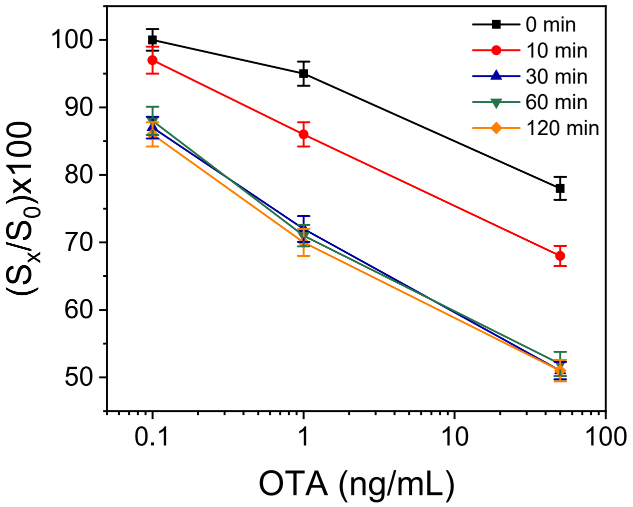

Figure S5. Effect of pre-incubation of anti-OTA antibody with OTA calibrators on assay sensitivity. Calibration curves obtained when the mixtures of the anti-OTA antibody with OTA calibrators were pre-incubated for 10 (red circles), 30 (blue up triangles), 60 (green down triangles) or 120 min (orange diamond). The calibration curve obtained without pre-incubation is also included (black squares). Each point is the mean value of three measurements ± SD.

Table S1. Zero calibrator signal values obtained from 12 measurements for the determination of SD and, consequently, assay LoD and LoQ.

Assay buffer	S_0 (nm)
Run 1	0.85
Run 2	0.82
Run 3	0.81
Run 4	0.81
Run 5	0.84

Run 6	0.81
Run 7	0.82
Run 8	0.81
Run 9	0.85
Run 10	0.84
Run 11	0.85
Run 12	0.85
Mean value ± SD	0.83 ± 0.02 (± 2.2 %)

Table S2. Zero calibrator signal values obtained from 8 wheat flour, 3 corn starch and 3 corn flour samples after extraction and 2-fold dilution.

**Cereal flour**	**S_0 (nm)**
Wheat flour 1	0.84
Wheat flour 2	0.84
Wheat flour 3	0.82
Wheat flour 4	0.84
Whole wheat flour 1	0.82
Whole wheat flour 2	0.83
Whole wheat flour 3	0.83
Whole wheat flour 4	0.84
Corn starch 1	0.85
Corn starch 2	0.85
Corn starch 3	0.82
Corn flour 1	0.84
Corn flour 2	0.83
Corn flour 3	0.83
Mean value ± SD	0.83±0.01

Table S3. Zero calibrator signal values obtained from 6 white wines and 6 red wines after 10-times dilution.

**Grape Variety**	**S_0 (nm)**
Savatiano 1	0.85
Savatiano 2	0.83
Moschofilero 1	0.82
Moschofilero 2	0.84
Assyrtiko 1	0.84
Assyrtiko 2	0.85
Xinomavro 1	0.83
Xinomavro 2	0.83
Merlot 1	0.82
Merlot 2	0.83
Syrah 1	0.85
Syrah 2	0.83
Mean value ± SD	0.84±0.01

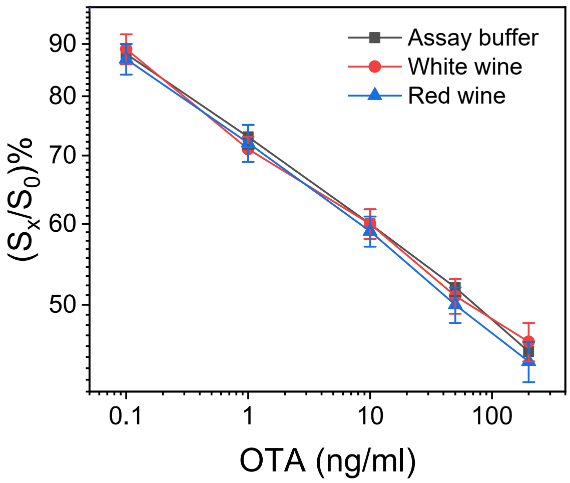

Figure S6. Calibration curves obtained with OTA calibrators prepared in assay buffer (black squares), white wine treated and 10-times diluted (red circles) or red wine treated and 10-times diluted (blue triangles). Each point is the mean value of three measurements ± SD.
